# The Potential Role of Polyphenols in Modulating Mitochondrial Bioenergetics within the Skeletal Muscle: A Systematic Review of Preclinical Models

**DOI:** 10.3390/molecules26092791

**Published:** 2021-05-10

**Authors:** Sinenhlanhla X. H. Mthembu, Phiwayinkosi V. Dludla, Khanyisani Ziqubu, Tawanda M. Nyambuya, Abidemi P. Kappo, Evelyn Madoroba, Thembeka A. Nyawo, Bongani B. Nkambule, Sonia Silvestri, Christo J. F. Muller, Sithandiwe E. Mazibuko-Mbeje

**Affiliations:** 1 Biomedical Research and Innovation Platform, South African Medical Research Council, Tygerberg 7505, South Africa; sinenhlanhla.Mthembu@mrc.ac.za (S.X.H.M.); Phiwayinkosi.dludla@mrc.ac.za (P.V.D.); Thembeka.Nyawo@mrc.ac.za (T.A.N.); christo.muller@mrc.ac.za (C.J.F.M.); 2 Department of Biochemistry and Microbiology, University of Zululand, KwaDlangezwa 3886, South Africa; MadorobaE@unizulu.ac.za; 3 Department of Biochemistry, Faculty of Natural and Agricultural Sciences, Mafikeng Campus, North West University, Private Bag X 2046, Mmabatho 2735, South Africa; ziqubukhanyisani@gmail.com; 4 Department of Health Sciences, Faculty of Health and Applied Sciences, Namibia University of Science and Technology, Windhoek 9000, Namibia; mnyambuya@nust.na; 5 School of Laboratory Medicine and Medical Sciences, College of Health Sciences, University of KwaZulu-Natal, Durban 4000, South Africa; nkambuleb@ukzn.ac.za; 6 Department of Biochemistry, Faculty of Science, Kingsway Campus, University of Johannesburg, Auckland Park 2006, South Africa; akappo@uj.ac.za; 7 Division of Medical Physiology, Faculty of Health Sciences, Stellenbosch University, Tygerberg 7505, South Africa; 8 Department of Life and Environmental Sciences, Polytechnic University of Marche, 60131 Ancona, Italy; s.silvestri@univpm.it

**Keywords:** polyphenols, skeletal muscle, mitochondrial function, insulin resistance, metabolic syndrome

## Abstract

Polyphenols are naturally derived compounds that are increasingly being explored for their various health benefits. In fact, foods that are rich in polyphenols have become an attractive source of nutrition and a potential therapeutic strategy to alleviate the untoward effects of metabolic disorders. The last decade has seen a rapid increase in studies reporting on the bioactive properties of polyphenols against metabolic complications, especially in preclinical models. Various experimental models involving cell cultures exposed to lipid overload and rodents on high fat diet have been used to investigate the ameliorative effects of various polyphenols against metabolic anomalies. Here, we systematically searched and included literature reporting on the impact of polyphenols against metabolic function, particularly through the modulation of mitochondrial bioenergetics within the skeletal muscle. This is of interest since the skeletal muscle is rich in mitochondria and remains one of the main sites of energy homeostasis. Notably, increased substrate availability is consistent with impaired mitochondrial function and enhanced oxidative stress in preclinical models of metabolic disease. This explains the general interest in exploring the antioxidant properties of polyphenols and their ability to improve mitochondrial function. The current review aimed at understanding how these compounds modulate mitochondrial bioenergetics to improve metabolic function in preclinical models on metabolic disease.

## 1. Introduction

Polyphenols are naturally derived compounds that are widely studied for their health benefits [1]. In fact, polyphenols can be grouped into four major categories, which include flavonoids, phenolic acids, stilbenes, and lignans. Flavonoids, one of the larger classes of polyphenols, can be further grouped into flavones, flavonols, flavanols, flavanones, isoflavones, proanthocyanidins, and anthocyanins [2]. Chemically, flavonoids have the universal structure of a 15-carbon skeleton, containing two phenyl rings and a heterocyclic ring. This carbon structure can be abbreviated as C6-C3-C6 [2]. Consumed food or beverage sources such as tea, fruits, and vegetables are known to contain high levels of polyphenols which include aspalathin, catechin, hesperetin, cyanidin, proanthocyanidins, quercetin, and rutin [3]. There is a significant interest in understanding the bioactivities of these compounds, with the PubMed search showing that over 4599 relevant records can be accessed to date, and a considerable growth in publications has been seen in the last decade [4]. In addition, many plants that are rich in polyphenols such as *Aspalathus linearis* “rooibos tea plant” [5] and *Camellia sinensis* “tea plant” [6] are widely investigated for their health benefits such as improving cardiovascular function and combating cancer [7,8]. In fact, our group has been actively involved in understanding the therapeutic effects of rooibos, including assessing its ameliorative effects against diverse metabolic complications [9,10]. Accumulatively, we have shown that polyphenolic compounds such as aspalathin, isoorientin, and rutin can activate various physiological pathways such as protein kinase B (AKT) and AMP-activated protein kinase (AMPK) to improve insulin signaling and regulate energy metabolism [11,12]. Likewise, polyphenolic compounds such as gallic acid and catechins can reduce body weight and attenuate metabolic abnormalities, especially scavenging free radical species through their abundant antioxidant properties [13].

Indeed, the bioactivity of polyphenols has been mainly attributed to their abundant antioxidant properties, which have been linked with improved metabolism, reduced inflammation, and ameliorating oxidative stress [14]. Notably, inflammation and oxidative stress are some of the key destructive components that are implicated in the development of metabolic anomalies and deteriorated metabolic health. Inflammation is characterized by enhanced pro-inflammatory cytokines such as tumor necrosis factor-α (TNF-α) and interleukin-6 (IL-6) [15]. On the other hand, oxidative stress arises because of overproduction of reactive oxygen species (ROS) that trigger suppression of intracellular antioxidant such as glutathione, superoxide dismutase, catalase, and thioredoxins [16]. Recently, impaired mitochondrial dysfunction has been reported to play an important role in the generation of oxidative stress through the altered actions of the electron transport chain [17]. For example, enhanced substrate delivery including free fatty acids (FFAs), especially under the conditions of metabolic syndrome, can impede the actions of the mitochondrial electron transport chain, resulting in the leakage of electrons and the overproduction of ROS. In fact, a few studies have correlated impaired mitochondrial bioenergetics with the generation of oxidative stress and reduced metabolic function [18]. As a result, many studies have targeted the main energy regulating tissues with abundant mitochondria, such as the skeletal muscle, to understand how increased substrate availability reduces or affects metabolic function [19,20]. Similarly, several studies have been published focusing on understanding how polyphenols affects mitochondrial bioenergetics in conditions of metabolic stress [21,22]. Currently, there is limited reviews on this topic or those targeting the modulation effect of polyphenols on skeletal muscle physiology. Thus, the current study aims to systematically extract and discuss relevant literature on the impact of polyphenols and plants rich in these compounds on their ameliorative effects against metabolic complications by targeting mitochondrial bioenergetics within the skeletal muscle.

## 2. Methodology for Study Selection and Inclusion

### 2.1. Data Sources and Search Strategies

The present review included preclinical trials obtained from a comprehensive search conducted on electronic databases, such as PubMed, from date of conception up to 30 December 2020. Two investigators, SXHM and KZ, independently conducted the search process and evaluated studies for eligibility and a third reviewer (PVD) was consulted in cases of disagreements. The systematic search was conducted using medical subject heading (MeSH) terms such as “polyphenols”, “bioactive compounds”, “mitochondria”, “metabolic syndrome”, and “skeletal muscle”. The search was restricted to English only. Mendeley reference manager version 1.19.4-dev2 software (Elsevier, Amsterdam, The Netherlands) was used to identify any duplicated studies.

### 2.2. Inclusion and Exclusion Criteria

This review includes in vitro and in vivo studies reporting on the impact of polyphenols on mitochondrial bioenergetics and related complications in skeletal muscle. In this review, only preclinical studies reporting on evidence involving skeletal muscle, polyphenols, and/or bioactive compounds and mitochondrial bioenergetics were included. This review is focused on better understanding the importance of polyphenols and bioactive compounds on pre-clinical studies, therefore human studies, books, letters, case reports, and reviews were excluded.

### 2.3. Data Extraction and Representation

Studies from the initial search on PubMed were screened for eligibility, they were then subsequently evaluated by full-text screening. Data was extracted by two investigators (SXHM and KZ) independently with (PVD) as a third investigator in case of any disagreements. Data extraction was performed in the following format: polyphenols/bioactive compounds, experimental model, effective dose, and intervention period, and main findings and author details (name and year of publication).

## 3. Results

### 3.1. An Overview of Results

**Figure 1:**
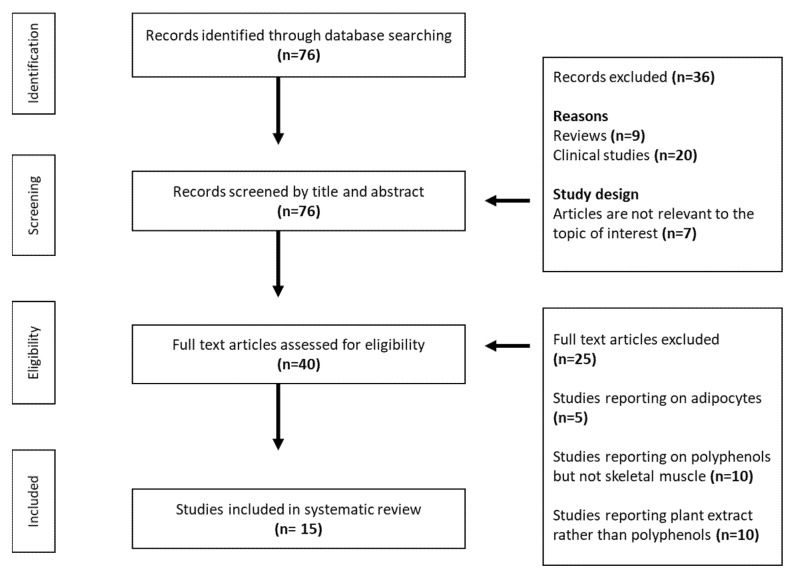
Flow chart representing the study selection procedure.

The primary outcome of the study was to evaluate the impact of polyphenols on mitochondrial bioenergetics, oxidative stress, and/or any other metabolic complications within the skeletal muscle. Figure 1 shows the flow chart of the study selection. Briefly, 7 studies were initially identified; however, after screening and reviewing the titles and abstracts, only 40 studies were eligible for the full-text assessment. After reviewing the full-text articles, a total of 25 studies were irrelevant to the topic of interest. Therefore, 15 met the inclusion criteria and were discussed within the review.

### 3.2. A Brief Overview on Polyphenolic Compounds and Their Impact on Mitochondrial Bioenergetics and Linked Metabolic Function in Various Preclinical Models

In addition to giving a brief background on the source and bioavailability profile, both in vivo and in vitro studies are discussed based on each polyphenolic compound, systematically extracted from the literature, as represented in Table 1 and Table 2.

#### 3.2.1. Resveratrol

**Figure 2:**
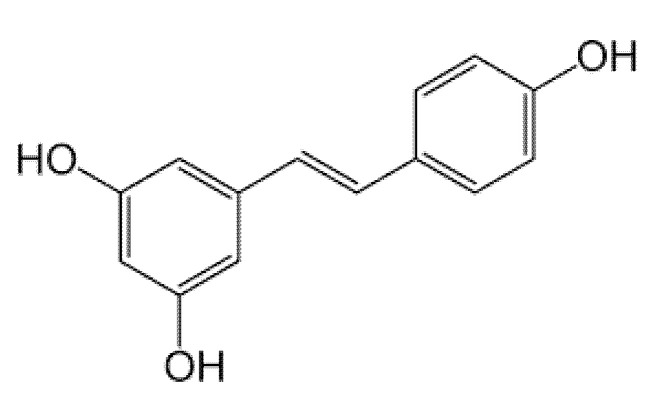
The chemical structure of resveratrol. A polyphenol commonly found in various plant food such as berries and wines [38].

Resveratrol (3,5,4′-trans-trihydroxystilbene, Figure 2) is a polyphenolic phytoalexin also belonging to the stilbene family that is abundant in grape skin and seeds, but is also found in various types of plant foods such as berries, peanuts, and wine [36]. This polyphenol is widely available and it is synthesized by more than 70 species of plants [37]. Although it exhibits low bioavailability and solubility [37], experimental data on resveratrol have been widely reviewed, and it has shown potential benefits for human health and exhibits protective effects against metabolic complications such as inflammation, oxidative stress, and aging. Moreover, resveratrol has shown promising properties in ameliorating complications linked with diseases such as diabetes and obesity. Evidence from studies by Price et al. [21] and Higashida et al. [23] demonstrated that resveratrol enhanced mitochondrial function and biogenesis in a SIRT1-dependent manner, and this was consistent with improved mtDNA content in palmitate-treated skeletal muscle cells and HFD-fed mice. This includes increasing the protein expression of PGC1α and other mitochondrial functional genes such as TFAM, mfn2, and drp1, as well as the activity of mitochondrial complexes I–V in skeletal muscle cells [21,23,24].

Furthermore, in vivo studies suggest that resveratrol exhibits strong antioxidant properties in improving skeletal muscle function in various HFD-induced insulin-resistant models [17,31,32]. These effects were shown by a decreased level of ROS, a strong indicator of oxidative stress, which occurred concomitant with restored antioxidant enzyme activities, including SOD, CAT, and GPx. Furthermore, Huang et al. [17] demonstrated that resveratrol ameliorated insulin resistance in HFD-induced obese Sprague Dawley rats by reducing intramuscular lipid accumulation and enhancing SIRT1 activity. This was, in part, by increasing mitochondrial biogenesis and β-oxidation in the skeletal muscle of these rats. More evidence included in this review demonstrated that resveratrol increased the phosphorylation of AMPK in the skeletal muscle of both C57/BL6J mice and HFD-induced sarcopenic obesity Sprague Dawley rats [17,21]. Overall, resveratrol demonstrates a wide array of benefits in improving metabolic function, in part by effectively regulating energy metabolism and mitochondrial bioenergetics within the skeletal muscle.

#### 3.2.2. Gingerol

**Figure 3:**
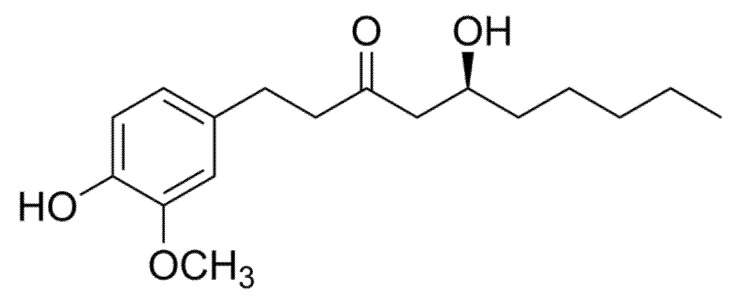
The chemical structure of gingerol. This polyphenol can be generally found as a pungent yellow oil in the ginger rhizome, which can be uniquely identified as a low-melting point crystalline solid [47].

Gingerol is the primary bioactive phenylpropanoid in the rhizome of ginger (*Z. officinale* Roscoe; Zingiberaceae) (Figure 3) which is known for its pungent taste and aroma [25]. Ginger is widely used a spice and medicinal herb, highlighting the general interest in the potential health benefits of the bioactive compounds found in this functional food product [39]. Generally, ginger contains pungent phenolic substances known as gingerols, shogaols, paradols, and zingerone [39]. Amongst the constituents of gingerols [6]-gingerol (1-[4′-hydroxy-3′-methoxyphenyl]-5-hydroxy-3-decanone) is the major pharmacologically active component [40,41]. This polyphenol is known to display a variety of biological properties, including anticancer [42], antioxidant, anti-inflammatory [43], and antifungal effects [44]. Our literature search showed that this bioactive compound has the potential to enhance mitochondrial function in L6 rat myotubes. Briefly, was is demonstrated that treating normal L6 myotubes with 50, 100, and 150 µM (S)-[6]-gingerol for 24 h could activate AMPKα and further improve mitochondrial content number and the gene expression of PGC1α in vitro [45]. Other studies reported that the polyphenol found in ginger could affect metabolic function by reducing blood glucose levels in diabetic animal models and increase glucose uptake in in vitro cultured cells [43,46]. Overall, (S)-[6]-gingerol displays the potential beneficial effects on metabolic function by modulating skeletal muscle mitochondrial function, further suggesting that ginger may be effective in preventing the development of metabolic syndromes. However, additional data is required to confirm its metabolic properties, there has been concern with regard to the low solubility and poor oral absorption of [6]-Gingerol, as reported elsewhere [42]

#### 3.2.3. Quercetin and Naringenin

**Figure 4:**
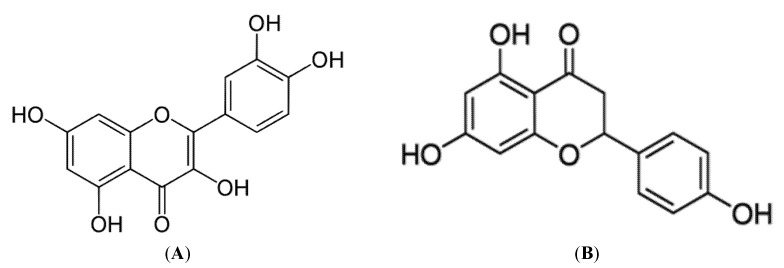
The chemical structure of quercetin (**A**) and naringenin (**B**). These polyphenols are predominant in vegetables, fruits, coffee, and tea in the form of a glycoside [53,54].

Quercetin (3,3′,4′,5,7-pentahydroxyflavone, Figure 4) has the ability to exhibit robust antioxidant, anti-apoptosis, and anti-inflammatory properties in different preclinical models [48]. For example, in our literature search, we found that quercetin also has the ability to enhance mitochondrial function [49]. Alternatively, Mutlur Krishnamoorthy, and Carani Venkatraman (2017) [26] showed that treating palmitate-induced insulin resistance L6 myotubes with 750 mM quercetin or 75 µM naringenin for 16 h could improve glucose homeostasis and mitochondrial bioenergetics by enhancing GLUT4 translocation, as well as increasing AMPK phosphorylation, and SIRT1 and PGC1α expression. Apparently, the comparative efficacy of quercetin and naringenin in ameliorating various metabolic anomalies has been subject to increasing preclinical research [22].

Naringenin (2,3-dihydro-5,7-dihydroxy-2-(4-hydroxyphenyl)-4H-1-benzopyran-4-one) is a naturally occurring flavonoid found mostly in some edible fruits, such as citrus species [50]. This flavonoid has been the subject of ongoing research to assess its broad biological effects in preclinical models. For example, this flavonoid has an ability to decrease some lipid peroxidation biomarkers and promote carbohydrate metabolism in preclinical models of metabolic syndrome [26]. Furthermore, naringenin has been shown to have antioxidant and anti-inflammatory effects [51]. A similar effect was observed in an in vivo study, where high fructose diet-induced insulin resistance Wister rats showed decreased mitochondrial function [52]. However, this effect was reversed in rats that were also fed 50 mg/kg body weight/day naringenin and quercetin for 6 weeks [26]. Here, both naringenin and quercetin reduced the plasma glucose and insulin levels, including GLUT4 translocation, as well as the expression of SIRT1, PGC1α, and AMPK phosphorylation in the insulin resistant Wister rats [26], suggesting that both these polyphenols may improve metabolic function in part by regulating energy metabolism, or by improving glucose uptake and targeting markers of mitochondrial function.

#### 3.2.4. Pinosylvin

**Figure 5:**
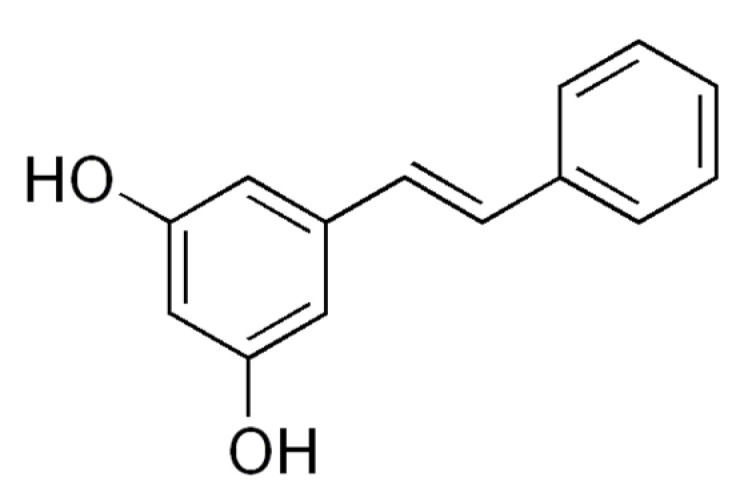
The chemical structure of pinosylvin, a stilbene-based compound found in plants, berries, and nuts [58].

Pinosylvin (3,5-dihydroxy-trans-stilbene, Figure 5) is part of the stilbenoids group, which is a group of polyphenols found in plants, berries, and nuts. These polyphenolic compounds exhibit antimicrobial and antifungal function in plants [55]. Recent information reveals that A stilbene-based compounds might have potential as antiviral agents [56]. Although the widely investigated naturally occurring stilbenoids such as resveratrol are acknowledged, on the other hand, emerging evidence suggests that pinosylvin is gaining attention due to it anti-inflammatory properties [57]. Pinosylvin is a natural polyphenol trans-stilbenoid that is produced by plants as a secondary metabolite to protect against microbes and insects [57]. This polyphenol is mainly found in heartwoods and leaves of *Pinus sylvestris*. Pinosylvin exerts various biological activities including anti-inflammatory effects [57]. In fact, Modi et al. [27] reported that treating cultured skeletal muscle cells (L6 myotube) with 20 or 60 µM pinosylvin for 24 h activated SIRT1 and stimulated glucose uptake through the activation of AMPK. Although the role of this stilbenoid is emerging, its effects on mitochondrial bioenergetics or function is still very limited.

#### 3.2.5. Icariin

**Figure 6:**
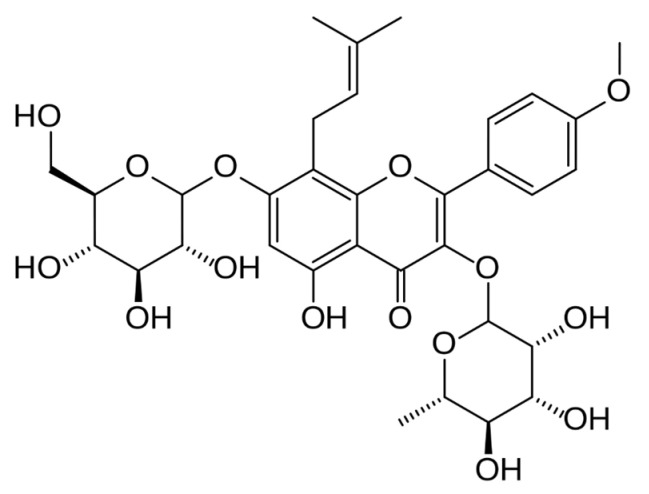
The chemical structure of icariin, which is commonly found in the extracts of Epimedii Herba. [63].

Icariin is a typical flavonol glycoside also known as the primary active component of Epimedii Herba (Figure 6) [28]. Icariin is commonly known as yin yang hou or goat weed [28]. The extracts of Epimedii Herba have been commonly used in Chinese herbal medicine to treat sexual functions, skeletal muscle deterioration, and other diseases [28,59]. Various pharmacological effects of icariin have been reported, including immunoregulation and vasodilation through the enhanced production of bioactive nitric oxide, as well as showing activity against multiple cardiovascular diseases through antioxidant and anti-inflammatory action [28,60]. In this review, we found that icariin might have a beneficial effect on the mitochondrial function [28], in part through effective modulation of energy metabolism related pathways/genes such as irisin/FNDC5, PGC1α gene expression, and dose-dependently increased AMPK phosphorylation in normal C2C12 cells. Interestingly, the same effect was also observed in C57BL/6 mice that were fed 10 or 40 mg/kg/day icariin for 14 days, displaying decreased body weight and enhanced expression of FNDC5, PGC1α, and p-AMPK levels. Other studies reported that icariin was also found to have a protective effect against diet-induced obesity by ameliorating insulin resistance [61,62]. Overall, the preclinical evidence summarized in this review seems to validate the anecdotal capacity of icariin to act on the skeletal muscle and modulate energy metabolism to potentially ameliorate metabolic disease related complications.

#### 3.2.6. Flavonoids, Flavanols and Proanthocyanidins

**Figure 7:**
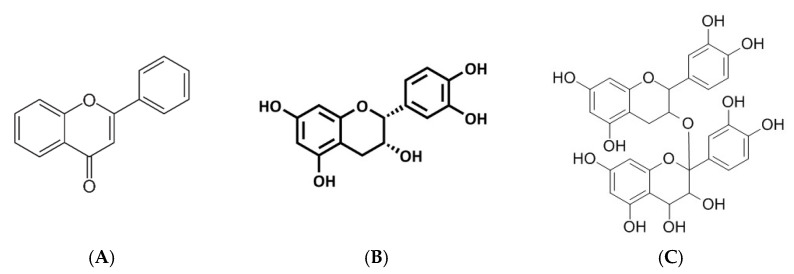
An overview of the chemical structure of flavonoids (**A**), flavanols (**B**), and proanthocyanidins (**C**). Briefly these polyphenols are commonly found in in plants and food sources such as wine, tea and chocolate [69,70].

Flavones and flavonols (Figure 7) are the most prominent ketone-containing compounds [64]. Furthermore, flavan-3-ols, also known as flavanols, are unique for containing the 2-phenyl-3,4-dihydro-2H-chromen-3-ol skeleton [65]. These compounds encompass catechin, epicatechin gallate, epigallocatechin, epigallocatechin gallate, proanthocyanidins, aflavins, and arubigins [2,66]. In fact, increasing literature has reported on the impact of these compounds in improving metabolic function in various preclinical models [33,67]. Reducing oxidative stress and inflammation, as well as regulating insulin signaling pathways such as the PI3K/AKT and energy homeostasis mechanisms such as the AMPK are the prominent effects by which these compounds may improve metabolic function [68].

Similarly, data from this review suggest that flavonoids from mulberry (*Morus alba* L.) leaves can perform the same as metformin (an established glucose lowering drug) in improving muscle glucose uptake and mitochondrial function in L6 muscle cells [31]. These actions were, achieved by activating AMPK and increasing the expression of PGC-1α and GLUT4 [31]. Importantly, the actions of these flavonoids were consistent with improved mitochondrial function in the skeletal muscle of *db*/*db* mice. Furthermore, flavan 3-ols fractions derived from cocoa powder were shown to promote lipolysis and mitochondrial biogenesis consistent with increasing β-oxidation through regulating carnitine palmitoyltransferase 2 (CPT2) expression and mitochondria copy number in mice with metabolic syndrome [35]. As one of the major flavonoids, proanthocyanidins were shown to improve skeletal muscle mitochondrial bioenergetics in obese Zucker fatty rats by reducing citrate synthase activity, oxidative phosphorylation complexes I and II levels, and Nrf1 gene expression, which in turn translated to ameliorated ROS production [33]. These actions were parallel to reduced insulin resistance, improved mitochondrial respiration, mitochondrial oxidative capacity, and fatty acid oxidation, with effective regulation of prominent energy regulation markers such as AMPK, Pparα, and UCP2 [33]. Overall, flavonoids and flavonols show great potential in improving metabolic function by effectively regulating skeletal muscle energy metabolism and mitochondrial bioenergetics in preclinical models of metabolic disease.

## 4. Summary and Future Perspective

It is now widely accepted that a healthy diet is essential to defend the human body against certain types of diseases, especially non-communicable diseases such as obesity, type 2 diabetes, and cardiovascular diseases [71]. Certainly, food sources such as fruits and vegetables have become an attractive source of nutrients and health benefits. In fact, these food sources are known to contain various biological compounds, including polyphenols, that present with enhanced potential beneficial effects in improving metabolic function. Accumulative preclinical evidence suggests that polyphenols can improve metabolic function by effectively regulating energy metabolism, as well as enhancing glucose uptake and mitochondrial function. Here, it was apparent that polyphenolic compounds such as gingerol, icariin, and resveratrol can target the skeletal muscle to regulate energy metabolism and improve mitochondrial function in preclinical models of metabolic syndrome. This is important to establish since it is already known that the pathogenesis of metabolic diseases like diabetes is consistent with skeletal muscle mitochondria deficiency, leading to impaired cellular functions [34,35,36]. Apparently, in addition to the effective modulation of cellular mechanisms such as insulin signaling and energy regulating pathways through PI3K/AKT and AMPK, these polyphenols seem to target PGC1α and other mitochondrial functional genes such as TFAM, mfn2, and drp1 to improve mitochondrial bioenergetics. These findings also highlight the potential impact naturally derived compounds and micronutrients can have on improving human health by targeting major organ tissues such as the skeletal muscle, as previously discussed [72]. In fact, the summarized data remain essential in developing precise therapeutic targets to be further tested in human subjects and to protect against the rapid rise of metabolic diseases. Although the current study informs on essential preclinical mechanisms that may be involved in the amelioration of metabolic complications, additional experiments and elucidations are still necessary to better understand the therapeutic potential of polyphenols, especially the relevance of their metabolism and bioavailability in the human body.

## Figures and Tables

**Table 1:** A summary of the in vitro studies reporting on the impact of polyphenols on mitochondrial bioenergetics and metabolic function within the skeletal muscle.

Polyphenols	Experimental Model	Effective Dose and Duration	Main Findings	Ref.
Resveratrol	C2C12 myoblast	25 µM resveratrol for 24 h	Enhanced mitochondrial function and biogenesis in a NAD-dependent deacetylase sirtuin-1 (SIRT1)-dependent manner. This included increasing ATP content and peroxisome proliferator-activated receptor γ coactivator 1-α (PGC1α) protein expression	[21]
	C2C12 myotubes	20 or 50 µM resveratrol for 6 or 24 h, respectively	High dose reduced ATP production and activated AMP-activated protein kinase (AMPK) phosphorylation. Resveratrol induced overexpression of SIRT1 decreased PGC1α acetylation and PGC1α coactivator activity	[23]
	C2C12 myoblast	20, 40, 60 μM resveratrol for 24 h	Increased miR-27b expression and mtDNA, which improved mitochondrial function and glucose uptake in a Sirt1-dependent manner	[24]
	Palmitate-induced mitochondrial dysfunction C2C12 myotubes	25 μM resveratrol for 24 h	Ameliorated mitochondrial dysfunction and oxidative stress as evident by improved mtDNA content and increased expression of mitochondrial biogenesis-r elated protein including PGC1α, mitochondrial transcription factor (TFAM), mitofusin 2 (mfn2), and drosophila melanogaster (drp1), as well as reduced ROS production	[17]
(S)-[6]-gingerol	L6 rat myotubes	50, 100 and 150 µM (S)-[6]-gingerol for 24 h	Activated AMPKα, which was accompanied by an increased mitochondrial content number, as well as an improved gene expression of PGC-1α	[25]
Naringenin and quercetin	Palmitate-induced insulin resistance L6 myotubes	75 µM naringenin or 750 mM quercetin for 16 h	Increased glucose transporter (GLUT)4 translocation, AMPK phosphorylation, and SIRT1 and PGC1α expression	[26]
Pinosylvin	Rats L6 myotubes	20 or 60 µM pinosylvin for 24 h	Pinosylvin activated SIRT1 in vitro and stimulated glucose uptake through the activation of AMPK	[27]
Icariin	C2C12 myocytes	20, 40, 80 μg/mL icariin for 24 h	Increased irisin/fibronectin type lll domain containing 5 (FNDC5), PGC1α gene expression, and dose-dependently increased AMPK phosphorylation	[28]
Flavonoids (mulberry.)	Palmitate-induced insulin resistance L6 myotubes	100 nmol/L insulin, 0.75 mmol/L Palmitic acid (PA) and MLF (5,10, 20, 40 and 80μg/mL) for 24 h	MLF and metformin significantly ameliorated glucose uptake by activating AMPK and reduced ROS production in L6 cells. Furthermore, MLF improved mitochondrial function by increasing the expression of PGC1α	[29]

**Table 2:** A summary of in vivo studies reporting on the impact of polyphenols on mitochondrial bioenergetics and metabolic function within the skeletal muscle.

Polyphenols	Experimental Model	Effective Dose and Duration	Main Findings	Ref.
Resveratrol	High-fat diet (HFD) induced obese C57BL/6J mice	400 mg/kg/day resveratrol for 15 weeks	Increased oxygen consumption was accompanied by regulation of the genes for mitochondrial biogenesis such as peroxisome proliferator-activated receptor γ coactivator 1 α (PGC1α) acetylation and activity	[30]
	HFD-fed Sprague Dawley rats	100 mg/kg b.w./day resveratrol for 8 weeks	Reduced intramuscular lipid accumulation and ameliorated insulin resistance, in part by enhancing NAD-dependent deacetylase sirtuin 1 (SIRT1) activity, increasing mitochondrial biogenesis and β-oxidation	[14]
	Catch-up growth-induced insulin resistance Sprague Dawley rats	100 mg/kg b.w./day resveratrol treatment for 4 and 8 weeks	Enhanced SIRT1 activity and improved mitochondrial number and insulin sensitivity, as well as decreased levels of reactive oxygen species and restored antioxidant enzyme activities, including superoxide dismutase (SOD), catalase (CAT), and glutathione peroxidase (GPx)	[31]
	C57/BL6J mice	25–30 mg/kg b.w/day (low dose) and 215–230 mg/kg b.w/day (high dose) resveratrol for 8 months	50 μM dose significantly decreased ATP levels early as 1 h after treatment and activated AMPK independently of SIRT1. At 25 µM resveratrol increased mitochondrial function by increased expression of PGC1α, PGC1β, and TFAM including the transcription factor B2 (TFB2M) in a SIRT1-dependent manner. This was also supported by an increase on mtDNA content. Furthermore, resveratrol AMP-activated protein kinase (AMPK) and increased NAD+ levels	[21]
	HFD-induced insulin resistance Sprague Dawley rats	100 mg/kg/day resveratrol for 8 weeks	Ameliorated insulin resistance through increased SIRT1 and SIRT3 expressions and elevated mtDNA and mitochondrial biogenesis. This included enhancing mitochondrial antioxidant enzymes including SOD, CAT, and GPx	[32]
	HFD-fed C57BL/6J mice	0.02, 0.04, and 0.06% resveratrol for 12 weeks	Reduced the plasma insulin and glucose concentrations, which were accompanied by an increased miR-27b overexpression, which improved mitochondrial function in a Sirt1-dependent manner	[24]
	HFD-induced sarcopenic obesity Sprague Dawley rats	0.4% resveratrol for 20 weeks	Ameliorated mitochondrial dysfunction and oxidative stress via the serine–threonine kinase LKB1 (PKA/LKB1)/AMPK pathway. This was evident by increased activity of complexes I, II, and IV, and raised PGC1α, TFAM, and mfn2, as well as decreased drp1 expression. Moreover, there was an increase in the total antioxidative capability (T-AOC), SOD, GPx, MDA, and carbonyl protein	[17]
Proanthocyanidins	Obese Zucker fatty rats (*fa/fa*)	35 mg/kg b.w./day proanthocyanidins 68 days	Decreased citrate synthase activity and oxidative phosphorylation complexes I and II levels and Nrf1 gene expression, which in turn reduced reactive oxygen species (ROS) production	[33]
	Diet-induced obese Wistar rats	25 mg/kg b.w./day proanthocyanidins for 21 days	Reduced insulin resistance, improved mitochondrial respiration, mitochondrial oxidative capacity, and fatty acid oxidation as evident by increased mitochondrial enzymatic activities, AMPK phosphorylation, and the expression of peroxisome proliferator-activated receptor α (*Pparα*) and UCP2	[34]
Flavan 3-ols fraction derived from cocoa powder	C57BL/J mice	50 mg/kg b.w./day flavan-3-ols for 2 weeks	Enhanced lipolysis and promoted mitochondrial biogenesis marked by increased carnitine palmitoyltransferase 2 (CPT2) expression and mitochondria copy number	[35]
Naringenin and quercetin	High-fructose diet-induced insulin resistance Wistar rats	50 mg/kg b.w./day naringenin and quercetin for 6 weeks	Both naringenin and quercetin reduced the plasma glucose and insulin levels accompanied by a significant increase in SIRT1 and PGC1α expression, AMPK phosphorylation, and glucose transporter type 4 (GLUT4) translocation	[26]
Icariin	C57BL/6 mice	10 or 40 mg/kg/day icariin for 14 days	Decrease in body weight gain by increasing FNDC5, PGC-1α, and p-AMPK expression levels	[28]
Flavonoids	Type 2 diabetic (*db*/*db*) mice	180 mg/kg flavonoids for 7 weeks	Ameliorated insulin resistance and symptoms associated with diabetes through increased p-AMPK and PGC1α, raised m-GLUT4 and T-GLUT4 protein expression, and improved mitochondrial function	[29]

## Data Availability

Data related to search strategy, study selection and extraction items will be made available upon request after the manuscript is published.

## Sample Availability

Samples of the compounds are available from the authors.

## Abbreviations

AKT: protein kinase B; AMPK, 5′ AMP-activated protein kinase; ATP, adenosine triphosphate; CAT, catalase; drp1, drosophila melanogaster; FFA, free fatty acids; HFD, high fat diet; GLUT 4, glucose transporter type 4; mfn2, mitofusin 2; mtDNA, mitochondrial DNA content; Nrf 1, nuclear factor erythroid-related factor 1; PGC1α, peroxisome proliferator-activated receptor γ coactivator 1 α; PI3K, phosphoinositide 3-kinase; ROS, reactive oxygen species; UCP-2, uncoupling protein-2; SIRT 1, sirtuin 1; SOD 1, superoxide dismutase 1; TFAM, mitochondrial transcription factor; GPx, glutathione peroxidase.

## References

[1] Fraga C.G., Croft K.D., Kennedy D.O., Tomás-Barberán F.A. (2019). The Effects of Polyphenols and Other Bioactives on Human Health. Food Funct..

[2] Panche A.N., Diwan A.D., Chandra S.R. (2016). Flavonoids: An overview. J. Nutr. Sci..

[3] Pandey K.B., Rizvi S.I. (2009). Plant Polyphenols as Dietary Antioxidants in Human Health and Disease. Oxid. Med. Cell. Longev..

[4] Di Lorenzo C., Colombo F., Biella S., Stockley C., Restani P. (2021). Polyphenols and Human Health: The Role of Bioavailability. Nutrients.

[5] Muller C.J.F., Malherbe C.J., Chellan N., Yagasaki K., Miura Y., Joubert E. (2018). Potential of rooibos, its major C-glucosyl flavonoids, and Z-2-(β-D-glucopyranosyloxy)-3-phenylpropenoic acid in prevention of metabolic syndrome. Crit. Rev. Food Sci. Nutr..

[6] Yu X., Xiao J., Chen S., Yu Y., Ma J., Lin Y., Li R., Lin J., Fu Z., Zhou Q. (2020). Metabolite signatures of diverse Camellia sinensis tea populations. Nat. Commun..

[7] Marnewick J.L., Rautenbach F., Venter I., Neethling H., Blackhurst D.M., Wolmarans P., MacHaria M. (2011). Effects of rooibos (Aspalathus linearis) on oxidative stress and biochemical parameters in adults at risk for cardiovascular disease. J. Ethnopharmacol..

[8] Beynon R.A., Richmond R.C., Santos Ferreira D.L., Ness A.R., May M., Smith G.D., Vincent E.E., Adams C., Ala-Korpela M., Würtz P. (2019). Investigating the effects of lycopene and green tea on the metabolome of men at risk of prostate cancer: The ProDiet randomised controlled trial. Int. J. Cancer.

[9] Dludla P.V., Johnson R., Mazibuko-Mbeje S.E., Muller C.J.F., Louw J., Joubert E., Orlando P., Silvestri S., Chellan N., Nkambule B.B. (2020). Fermented rooibos extract attenuates hyperglycemia-induced myocardial oxidative damage by improving mitochondrial energetics and intracellular antioxidant capacity. S. Afr. J. Bot..

[10] Mazibuko-Mbeje S.E., Dludla P.V., Johnson R., Joubert E., Louw J., Ziqubu K., Tiano L., Silvestri S., Orlando P., Opoku A.R. (2019). Aspalathin, a natural product with the potential to reverse hepatic insulin resistance by improving energy metabolism and mitochondrial respiration. PLoS ONE.

[11] Mazibuko-Mbeje S.E., Ziqubu K., Dludla P.V., Tiano L., Silvestri S., Orlando P., Nyawo T.A., Louw J., Kappo A.P., Muller C.J. (2020). Isoorientin ameliorates lipid accumulation by regulating fat browning in palmitate-exposed 3T3-L1 adipocytes. Metab. Open..

[12] Mazibuko-Mbeje S.E., Dludla P.V., Roux C., Johnson R., Ghoor S., Joubert E., Louw J., Opoku A.R., Muller C.J. (2019). Aspalathin-enriched green rooibos extract reduces hepatic insulin resistance by modulating PI3K/AKT and AMPK pathways. Int. J. Mol. Sci..

[13] Luo Q., Zhang J.-R., Li H.-B., Wu D.-T., Geng F., Corke H., Wei X.-L., Gan R.-Y. (2020). Green Extraction of Antioxidant Polyphenols from Green Tea (*Camellia sinensis*). Antioxidants.

[14] Chen L.L., Zhang H.H., Zheng J., Hu X., Kong W., Hu D., Wang S.X., Zhang P. (2011). Resveratrol attenuates high-fat diet-induced insulin resistance by influencing skeletal muscle lipid transport and subsarcolemmal mitochondrial β-oxidation. Metabolism.

[15] Lainampetch J., Panprathip P., Phosat C., Chumpathat N., Prangthip P., Soonthornworasiri N., Puduang S., Wechjakwen N., Kwanbunjan K. (2019). Association of Tumor Necrosis Factor Alpha, Interleukin 6, and C-Reactive Protein with the Risk of Developing Type 2 Diabetes: A Retrospective Cohort Study of Rural Thais. J. Diabetes Res..

[16] Truong V.-L., Jun M., Jeong W.-S. (2018). Role of resveratrol in regulation of cellular defense systems against oxidative stress. BioFactors.

[17] Huang Y., Zhu X., Chen K., Lang H., Zhang Y., Hou P., Ran L., Zhou M., Zheng J., Yi L. (2019). Resveratrol prevents sarcopenic obesity by reversing mitochondrial dysfunction and oxidative stress via the PKA/LKB1/AMPK pathway. Aging.

[18] Di Meo S., Iossa S., Venditti P. (2017). Skeletal muscle insulin resistance: Role of mitochondria and other ROS sources. J. Endocrinol..

[19] Coudray C., Fouret G., Lambert K., Ferreri C., Rieusset J., Blachnio-Zabielska A., Lecomte J., Ebabe Elle R., Badia E., Murphy M.P. (2016). A mitochondrial-targeted ubiquinone modulates muscle lipid profile and improves mitochondrial respiration in obesogenic diet-fed rats. Br. J. Nutr..

[20] Jørgensen W., Rud K.A., Mortensen O.H., Frandsen L., Grunnet N., Quistorff B. (2017). Your mitochondria are what you eat: A high-fat or a high-sucrose diet eliminates metabolic flexibility in isolated mitochondria from rat skeletal muscle. Physiol. Rep..

[21] Price N.L., Gomes A.P., Ling A.J.Y., Duarte F.V., Martin-Montalvo A., North B.J., Agarwal B., Ye L., Ramadori G., Teodoro J.S. (2012). SIRT1 is required for AMPK activation and the beneficial effects of resveratrol on mitochondrial function. Cell Metab..

[22] Sharma S., Tripathi P., Sharma J., Dixit A. (2020). Flavonoids modulate tight junction barrier functions in hyperglycemic human intestinal Caco-2 cells. Nutrition.

[23] Higashida K., Kim S.H., Jung S.R., Asaka M., Holloszy J.O., Han D.H. (2013). Effects of Resveratrol and SIRT1 on PGC-1α Activity and Mitochondrial Biogenesis: A Reevaluation. PLoS Biol..

[24] Zhou X., Zuo S., Xin W. (2015). miR-27b overexpression improves mitochondrial function in a Sirt1-dependent manner. J. Physiol. Biochem..

[25] Li Y., Tran V.H., Kota B.P., Nammi S., Duke C.C., Roufogalis B.D. (2014). Preventative effect of zingiber officinale on insulin resistance in a high-fat high-carbohydrate diet-fed rat model and its mechanism of action. Basic Clin. Pharmacol. Toxicol..

[26] Mutlur Krishnamoorthy R., Carani Venkatraman A. (2017). Polyphenols activate energy sensing network in insulin resistant models. Chem. Biol. Interact..

[27] Modi S., Yaluri N., Kokkola T., Laakso M. (2017). Plant-derived compounds strigolactone GR24 and pinosylvin activate SIRT1 and enhance glucose uptake in rat skeletal muscle cells. Sci. Rep..

[28] Chen S.Q., Ding L.N., Zeng N.X., Liu H.M., Zheng S.H., Xu J.W., Li R.M. (2019). Icariin induces irisin/FNDC5 expression in C2C12 cells via the AMPK pathway. Biomed. Pharmacother..

[29] Meng Q., Qi X., Fu Y., Chen Q., Cheng P., Yu X., Sun X., Wu J., Li W., Zhang Q. (2020). Flavonoids extracted from mulberry (*Morus alba* L.) leaf improve skeletal muscle mitochondrial function by activating AMPK in type 2 diabetes. J. Ethnopharmacol..

[30] Lagouge M., Argmann C., Gerhart-Hines Z., Meziane H., Lerin C., Daussin F., Messadeq N., Milne J., Lambert P., Elliott P. (2006). Resveratrol Improves Mitochondrial Function and Protects against Metabolic Disease by Activating SIRT1 and PGC-1α. Cell.

[31] Zheng J., Chen L.L., Zhang H.H., Hu X., Kong W., Hu D. (2012). Resveratrol improves insulin resistance of catch-up growth by increasing mitochondrial complexes and antioxidant function in skeletal muscle. Metabolism.

[32] Haohao Z., Guijun Q., Juan Z., Wen K., Lulu C. (2015). Resveratrol improves high-fat diet induced insulin resistance by rebalancing subsarcolemmal mitochondrial oxidation and antioxidantion. J. Physiol. Biochem..

[33] Pajuelo D., Fernández-Iglesias A., Díaz S., Quesada H., Arola-Arnal A., Bladé C., Salvadó J., Arola L. (2011). Improvement of mitochondrial function in muscle of genetically obese rats after chronic supplementation with proanthocyanidins. J. Agric. Food Chem..

[34] Casanova E., Baselga-Escudero L., Ribas-Latre A., Cedó L., Arola-Arnal A., Pinent M., Bladé C., Arola L., Salvadó M.J. (2014). Chronic intake of proanthocyanidins and docosahexaenoic acid improves skeletal muscle oxidative capacity in diet-obese rats. J. Nutr. Biochem..

[35] Watanabe N., Inagawa K., Shibata M., Osakabe N. (2014). Flavan-3-Ol Fraction from Cocoa Powder Promotes Mitochondrial Biogenesis in Skeletal Muscle in Mice. Lipids Health Dis..

[36] Shrikanta A., Kumar A., Govindaswamy V. (2015). Resveratrol content and antioxidant properties of underutilized fruits. J. Food Sci. Technol..

[37] Marier J.F., Vachon P., Gritsas A., Zhang J., Moreau J.P., Ducharme M.P. (2002). Metabolism and disposition of resveratrol in rats: Extent of absorption, glucuronidation, and enterohepatic recirculation evidenced by a linked-rat model. J. Pharmacol. Exp. Ther..

[38] Resveratrol|CAS:501-36-0|Price: $30/20 mg|Manufacturer ChemFaces. http://www.chemfaces.com/natural/Resveratrol-CFN98791.html.

[39] Wang Q., Wei Q., Yang Q., Cao X., Li Q., Shi F., Tong S.S., Feng C., Yu Q., Yu J. (2018). A novel formulation of [6]-gingerol: Proliposomes with enhanced oral bioavailability and antitumor effect. Int. J. Pharm..

[40] Xu Y., Wang Q., Feng Y., Firempong C.K., Zhu Y., Omari-Siaw E., Zheng Y., Pu Z., Xu X., Yu J. (2016). Enhanced oral bioavailability of [6]-Gingerol-SMEDDS: Preparation, in vitro and in vivo evaluation. J. Funct. Foods.

[41] Madkor H.R., Mansour S.W., Ramadan G. (2010). Modulatory effects of garlic, ginger, turmeric and their mixture on hyperglycaemia, dyslipidaemia and oxidative stress in streptozotocin-nicotinamide diabetic rats. Br. J. Nutr..

[42] Lin C.B., Lin C.C., Tsay G.J. (2012). 6-gingerol inhibits growth of colon cancer cell LoVo via induction of G2/M arrest. Evid. Based Complement. Altern. Med..

[43] Dugasani S., Rao Pichika M., Nadarajah V.D., Katyayani Balijepalli M., Tandra S., Narsimha Korlakunta J. (2010). Comparative antioxidant and anti-inflammatory effects of [6]-gingerol, [8]-gingerol, [10]-gingerol and [6]-shogaol. J. Ethnopharmacol..

[44] Sharma S., Yadav A. (2020). Gingerol Derivatives as 14α-demethylase Inhibitors: Design and Development of Natural, Safe Antifungals for Immune-compromised Patients. Lett. Drug Des. Discov..

[45] Li Y., Tran V., Duke C., Roufogalis B. (2012). Gingerols of Zingiber officinale Enhance Glucose Uptake by Increasing Cell Surface GLUT4 in Cultured L6 Myotubes. Planta Med..

[46] Samad M.B., Mohsin M.N.A.B., Razu B.A., Hossain M.T., Mahzabeen S., Unnoor N., Muna I.A., Akhter F., Kabir A.U., Hannan J.M.A. (2017). [6]-Gingerol, from Zingiber officinale, potentiates GLP-1 mediated glucose-stimulated insulin secretion pathway in pancreatic β-cells and increases RAB8/RAB10-regulated membrane presentation of GLUT4 transporters in skeletal muscle to improve hyperglycemia in Leprdb/db type 2 diabetic mice. BMC Complement. Altern. Med..

[47] 6-Gingerol|CAS:23513-14-6|Price: $80/20 mg|Manufacturer ChemFaces. http://www.chemfaces.com/natural/6-Gingerol-CFN99931.html.

[48] Alrawaiq N.S., Abdullah A. (2014). A review of flavonoid quercetin: Metabolism, bioactivity and antioxidant properties. Int. J. Pharm Tech Res..

[49] Ahmed O.M., Ahmed A.A., Fahim H.I., Zaky M.Y. (2019). Quercetin and naringenin abate diethylnitrosamine/acetylaminofluorene-induced hepatocarcinogenesis in Wistar rats: The roles of oxidative stress, inflammation and cell apoptosis. Drug Chem. Toxicol..

[50] Tu B., Liu Z.J., Chen Z.F., Ouyang Y., Hu Y.J. (2015). Understanding the structure-activity relationship between quercetin and naringenin: In vitro. RSC Adv..

[51] Ke J.Y., Banh T., Hsiao Y.H., Cole R.M., Straka S.R., Yee L.D., Belury M.A. (2017). Citrus flavonoid naringenin reduces mammary tumor cell viability, adipose mass, and adipose inflammation in obese ovariectomized mice. Mol. Nutr. Food Res..

[52] Alam M., Kauter K., Brown L. (2013). Naringin Improves Diet-Induced Cardiovascular Dysfunction and Obesity in High Carbohydrate, High Fat Diet-Fed Rats. Nutrients.

[53] Quercetin|CAS:117-39-5|Price: $40/20 mg|Manufacturer ChemFaces. http://www.chemfaces.com/natural/Quercetin-CFN99272.html.

[54] Naringenin|CAS:480-41-1|Price: $30/20 mg|Manufacturer ChemFaces. http://www.chemfaces.com/natural/Naringenin-CFN98742.html.

[55] Eräsalo H., Hämäläinen M., Leppänen T., Mäki-Opas I., Laavola M., Haavikko R., Yli-Kauhaluoma J., Moilanen E. (2018). Natural Stilbenoids Have Anti-Inflammatory Properties in Vivo and Down-Regulate the Production of Inflammatory Mediators NO, IL6, and MCP1 Possibly in a PI3K/Akt-Dependent Manner. J. Nat. Prod..

[56] Wahedi H.M., Ahmad S., Abbasi S.W. (2020). Stilbene-based natural compounds as promising drug candidates against COVID-19. J. Biomol. Struct. Dyn..

[57] Pinosylvin|CAS:22139-77-1|Price: $288/20 mg|Manufacturer ChemFaces. http://www.chemfaces.com/natural/Pinosylvin-CFN98203.html.

[58] Pecyna P., Wargula J., Murias M., Kucinska M. (2020). More Than Resveratrol: New Insights into Stilbene-Based Compounds. Biomolecules.

[59] Wang Y., Wang Y.S., Song S.L., Liang H., Ji A.G. (2016). Icariin inhibits atherosclerosis progress in Apoe null mice by downregulating CX3CR1 in macrophage. Biochem. Biophys. Res. Commun..

[60] Hu Y., Sun B., Liu K., Yan M., Zhang Y., Miao C., Ren L. (2016). Icariin Attenuates High-cholesterol Diet Induced Atherosclerosis in Rats by Inhibition of Inflammatory Response and p38 MAPK Signaling Pathway. Inflammation.

[61] Han Y., Jung H.W., Park Y.K. (2015). Effects of Icariin on insulin resistance via the activation of AMPK pathway in C2C12 mouse muscle cells. Eur. J. Pharmacol..

[62] Icariin|CAS:489-32-7|Price: $30/20 mg|Manufacturer ChemFaces. http://www.chemfaces.com/natural/Icariin-CFN99554.html.

[63] Han L.Y., Wu Y.L., Zhu C.Y., Wu C.S., Yang C.R. (2019). Improved pharmacokinetics of icariin (ica) within formulation of peg-plla/pdla-pnipam polymeric micelles. Pharmaceutics.

[64] Raman G., Shams-White M., Avendano E.E., Chen F., Novotny J.A., Cassidy A. (2018). Dietary intakes of flavan-3-ols and cardiovascular health: A field synopsis using evidence mapping of randomized trials and prospective cohort studies. Syst. Rev..

[65] Lee M.K., Kim H.W., Lee S.H., Kim Y.J., Asamenew G., Choi J., Lee J.W., Jung H.A., Yoo S.M., Kim J.B. (2019). Characterization of catechins, theaflavins, and flavonols by leaf processing step in green and black teas (Camellia sinensis) using UPLC-DAD-QToF/MS. Eur. Food Res. Technol..

[66] Oliveira J., Mateus N., de Freitas V. (2013). Flavanols: Catechins and proanthocyanidins. Natural Products: Phytochemistry, Botany and Metabolism of Alkaloids, Phenolics and Terpenes.

[67] Legeay S., Rodier M., Fillon L., Faure S., Clere N. (2015). Epigallocatechin Gallate: A Review of Its Beneficial Properties to Prevent Metabolic Syndrome. Nutrients.

[68] Flavone|CAS:525-82-6|Price: $30/20 mg|Manufacturer ChemFaces. http://www.chemfaces.com/natural/Flavone-CFN70130.html.

[69] Proanthocyanidins|CAS:4852-22-6|Price: $70/20 mg|Manufacturer ChemFaces. http://www.chemfaces.com/natural/Proanthocyanidins-CFN99556.html.

[70] Nair H.B., Sung B., Yadav V.R., Kannappan R., Chaturvedi M.M., Aggarwal B.B. (2010). Delivery of antiinflammatory nutraceuticals by nanoparticles for the prevention and treatment of cancer. Biochem. Pharmacol..

[71] Koch W. (2019). Dietary polyphenols-important non-nutrients in the prevention of chronic noncommunicable diseases. A systematic review. Nutrients.

[72] Moretti A., Paoletta M., Liguori S., Bertone M., Toro G., Iolascon G. (2020). Choline: An Essential Nutrient for Skeletal Muscle. Nutrients.

